# Low‐copy nuclear markers in *Isoëtes* (Isoëtaceae) identified with transcriptomes

**DOI:** 10.1002/aps3.1142

**Published:** 2018-05-08

**Authors:** Peter W. Schafran, Gabriel Johnson, W. Carl Taylor, Elizabeth A. Zimmer, Lytton J. Musselman

**Affiliations:** ^1^ Department of Biological Sciences Old Dominion University Norfolk Virginia 23529 USA; ^2^ Department of Botany National Museum of Natural History Smithsonian Institution Washington D.C. 20560 USA

**Keywords:** *gapC*, *IBR3*, *Isoëtes*, *pgiC*, primer design, Sanger sequencing

## Abstract

**Premise of the Study:**

Few genetic markers provide phylogenetic information in closely related species of *Isoëtes* (Isoëtaceae). We describe the development of primers for several putative low‐copy nuclear markers to resolve the phylogeny of *Isoëtes*, particularly in the southeastern United States.

**Methods and Results:**

We identified regions of interest in *Isoëtes* transcriptomes based on low‐copy genes in other plants. Primers were designed for these regions and tested with 16 taxa of *Isoëtes* and one species of *Lycopodium*. Parts of the *pgiC*,* gapC*, and *IBR3* gene regions show phylogenetic signal within the North American and Mediterranean clades of *Isoëtes*.

**Conclusions:**

Transcriptome data prove useful for identification and primer design of low‐copy genes. Three new markers show potential for inferring phylogenies in regional clades of *Isoëtes*, and possibly across the entire genus.

*Isoëtes* L. (Isoëtaceae, Lycopodiophyta) is a cosmopolitan genus of ca. 250 recognized species. These heterosporous lycophytes consist of a 2–3‐lobed rootstock that bears linear, quill‐like, microphyllous leaves or sporophylls. All microphylls have the potential to develop into sporophylls (Foster and Gifford, 1974). Mega‐ and microsporangia are produced at the base of sporophylls, in some species covered by a layer of tissue called a velum. Traditionally, spore ornamentation and velum coverage have been considered taxonomically important. Although species inhabit a variety of ecological niches, from obligate aquatic to ephemeral terrestrial habitats, their morphology is extremely conserved. Phylogenetic studies in closely related clades of *Isoëtes* have been limited by a dearth of morphological features and molecular markers. Hoot and Taylor (2001) identified the nuclear ribosomal gene internal transcribed spacer (ITS), a *LEAFY* homolog nuclear gene intron (*LFY*), and the plastid *atpB‐rbcL* spacer region as informative markers in *Isoëtes*. However, although these markers and the plastid *rbcL* gene show utility in large‐scale, global phylogenies, they generally lose resolution at the regional level (Rydin and Wikström, 2002; Hoot et al., 2006; Larsén and Rydin, 2016). *LFY* is more variable than the other three markers and is fairly informative in recently diverged species groups (Taylor et al., 2004; Hoot et al., 2004). With only a single informative nuclear marker within groups such as the eastern North American clade, it is difficult to fully test phylogenetic hypotheses of reticulate evolution and incomplete lineage sorting.

Transcriptomes provide a valuable tool for marker selection and PCR primer design in the absence of a sequenced genome, as is the case in *Isoëtes*. Databases such as the 1000 Plants project (http://www.onekp.com; Matasci et al., 2014) contain transcriptomes across all major lineages of land plants, allowing identification of unique marker regions for a group of interest. Here we describe use of transcriptome data to develop PCR primers for phylogenetically informative low‐copy nuclear markers in *Isoëtes*.

## METHODS AND RESULTS

Markers of interest were selected based on a literature search of reportedly low‐copy nuclear markers in ferns and mosses (Table 1; Szövényi et al., 2006; Schuettpelz et al., 2008; Rothfels et al., 2013). Nucleotide sequences for these markers were obtained from the National Center for Biotechnology Information's (NCBI) GenBank (http://www.ncbi.nlm.nih.gov/genbank/; Clark et al., 2016) or TreeBASE (http://www.treebase.org; Sanderson et al., 1994) databases. Transcriptomes for three *Isoëtes* taxa were provided by other sources (*I. echinospora* Durieu from S. Hetherington, University of Oxford, Oxford, United Kingdom; and *I. tegetiformans* Rury and an unnamed *Isoëtes* species from the 1000 Plants project [http://www.oneKP.com]). Using the BLAST+ 2.4 software package (Camacho et al., 2009), local BLAST databases were constructed from each *Isoëtes* transcriptome. The sequences of selected fern (Rothfels et al., 2013) and moss (Szövényi et al., 2006) low‐copy nuclear markers were BLASTed against the transcriptome databases to identify those markers present as single‐copy in *Isoëtes*. These single‐copy marker regions were extracted from their respective transcriptome and aligned with marker sequences from the literature using Geneious version 7 (Kearse et al., 2012). Primer sequences from the literature were modified to match the *Isoëtes* transcriptome sequences.

**Table 1:** Primers designed for low‐copy markers identified in *Isoëtes* transcriptomes

Marker ID	Primer names	Primer sequences (5′–3′)	*T*_a_ (°C)
pgiC	pgiC_1156F pgiC_1900R	F: GGTCTCCTAAGTGTCTGGAATGT	55
		R: GTTCTCCAAAATCAATTTCTCC	
IBR3_1	IBR3_2F IBR3_6R	F: CTCAAATCAGCTCATGCAATTG	60
		R: AGCTCCCAATCCAACACAGC	
IBR3_2	IBR3_13F IBR3_16R	F: CAATGACTGAACCGCAAGTTG	60
		R: GACCCAACGAGTCTCATGCAG	
Transducin_1	Transducin_1F Transducin_1R	F: GATGTGGTTGGTGAGTCTGG	55
		R: CACTTCATTGAACCTCAG	
Transducin_2	Transducin_2F Transducin_2R	F: GGAACAAAAGCAGGGACATTAG	55
		R: CATCAGAAGAGATGTCCATAC	
gapC_short	gapC_5F gapC_7R	F: GAATCTACTGGTGTCTTCAC	55
		R: TTCTGGTTTATATTCATGCTCG	
gapC_long	gapC_5F gapC_9R	F: GAATCTACTGGTGTCTTCAC	55
		R: ATGGTCCATCAACAGTYTTCTG	

Note

F = forward; R = reverse; *T*_a_ = annealing temperature.

Plants were collected from the field, and leaf tissue was desiccated with silica gel. Voucher specimens have been stored at the Old Dominion University herbarium (ODU) and/or the U.S. National Herbarium (US). DNA was extracted from approximately 200 mg of dried tissue with the QIAGEN DNeasy Plant Mini Kit (QIAGEN Inc., Valencia, California, USA) or AutogenPrep 965 (Autogen Inc., Holliston, Mississippi, USA) using standard protocols. Sixteen diploid taxa of *Isoëtes* and one species of *Lycopodium* L. (one individual per taxon) were selected from available DNAs to represent various levels of divergence (Appendix 1).

Markers were amplified by PCR on an ABI 2720 thermocycler (Thermo Fisher Scientific Inc., Waltham, Massachusetts, USA), with a reaction mixture of 12.5 μL of 2× GoTaq PCR master mix (Promega Corporation, Madison, Wisconsin, USA), 0.5 μL of 0.1 mg/mL bovine serum albumin, 1.0 μL each of 10 μM forward and reverse primer, 7.5 μL of sterile distilled water, and 2.5 μL of DNA template (10–60 ng). PCR reactions were carried out with an initial melting period at 94°C (5 min), followed by 35 cycles of 94°C (30 s), annealing at 55–60°C (30 s), and extension at 72°C (1 min), with a final extension at 72°C (7 min). Amplification success was confirmed by electrophoresis using a 1.5% sodium boric acid–based agarose gel.

Markers were selected for Sanger sequencing based on their producing a single band across all samples and for a maximum size of ~1000 bp. PCR products were treated with ExoSAP‐IT PCR cleanup enzyme mix (Affymetrix Inc., Santa Clara, California, USA) before cycle sequencing with BigDye Terminator v3.1 (Thermo Fisher Scientific Inc.). The labeled sequencing fragments were read on an ABI 3130xl Genetic Analyzer (Thermo Fisher Scientific Inc.), and the resulting chromatograms were edited and analyzed using Geneious (Kearse et al., 2012).

Initial screening of primers showed that all amplify in at least some of the eastern North American taxa. Gel electrophoresis revealed that IBR3_1 and Transducin_2 are too long (~2000 bp) and Transducin_1 has both short and long copies in some individuals (~500 bp and ~1000 bp), making these poor candidates for a Sanger sequencing approach without needing molecular cloning or gel extraction. Although gapC_short readily amplified, it is contained within gapC_long, making sequencing of the shorter fragment redundant. pgiC, IBR3_2 (hereafter IBR3), and gapC_long (hereafter gapC) were selected for PCR and sequencing of the full taxa list (Appendices 2, 3).

### pgiC

This primer pair is rooted in exons 14 and 16, and amplifies across introns 14, 15, and exon 15 of this locus (Rothfels et al., 2013). The region amplified easily across all taxa of *Isoëtes* and *Lycopodium clavatum* L., and generated consistently high‐quality sequence data. All sequences aligned well, with a total alignment length of 466 bp and pairwise identity of 83%. Excluding *L. clavatum*, alignment length decreases to 357 bp and pairwise identity increases to 89%. Sequence length between these species of *Isoëtes* ranges from 310 to 347 bp, with a mean of 324 bp (Table 2). This is approximately half the length of the same region in ferns tested by Rothfels et al. (2013).

**Table 2:** Alignment statistics for all sequences with quality scores >85%

Marker	*Isoëtes*					*Lycopodium* + *Isoëtes*				
	Amplicon length range, bp (Mean)	Alignment length, bp	Pairwise % identity	No. of identical sites (%)	No. of PIS (%)	Amplicon length range, bp (Mean)	Alignment length, bp	Pairwise % identity	No. of identical sites (%)	No. of PIS (%)
pgiC	310–347 (324)	357	89	240 (67)	80 (22)	310–458 (331)	466	83	192 (41)	82 (18)
IBR3	587–682 (659)	700	87	415 (59)	111 (16)	—	—	—	—	—
gapC	443–543 (507)	561	85	304 (54)	95 (17)	—	—	—	—	—

Note

PIS = parsimony informative sites.

### gapC

The *gapC* gene encodes cytosolic glyceraldehyde‐3‐phosphate and is part of the GAPDH gene family (Strand et al., 1997; Wall, 2002; Szövényi et al., 2006). Primers designed by Szövényi et al. (2006) are rooted in exons 5 and 9 and amplify all exons and introns in between. However, given concern that the resulting marker in *Isoëtes* may be too long for Sanger sequencing, the primers designed for this study were rooted in exons 5 and 8, amplifying introns 5, 6, 7, and exons 6 and 7.

This marker showed the least ability to routinely generate high‐quality sequence data. Although not detected in any of the transcriptomes available, it is possible this results from off‐target amplification of other members of the GADPH gene family (i.e., *gapCp* or an unnamed *gapC*/*gapCp* relative) (Schuettpelz et al., 2008; Rothfels et al., 2013). The *Isoëtes*‐only alignment is 561 bp and has a pairwise identity of 85% (Table 2).

### IBR3

Unlike *pgiC* and *gapC*, this gene does not have an extensive history of use as a phylogenetic marker. The *IBR3* gene is thought to encode an indole‐3‐butyric acid–specific peroxisomal enzyme related to acyl‐CoA dehydrogenases (Zolman et al., 2007). Rothfels et al. (2013) showed it to be single‐copy throughout selected fern lineages, and this also appears to be the case in *Isoëtes*. Primers for the IBR3 marker amplify most species of *Isoëtes* easily, with the exception of two members of the Mediterranean clade (*I. histrix* Bory & Durieu and *I. nuttallii* A. Braun ex Engelm.). Alignment of *Isoëtes* sequences is 700 bp long with 87% pairwise identity (Table 2).

## CONCLUSIONS

Transcriptome mining is shown to be a useful tool for identification of putative low‐copy markers for primer design. Despite having access to transcriptomes of just three species of *Isoëtes* in the North American clade, primers could be designed for regions that show phylogenetic signal across widely divergent clades in the genus, and potentially across all Lycopodiophyta. Although techniques such as target enrichment allow for generation of data sets orders of magnitude larger (Mandel et al., 2014), design of primers for Sanger sequencing is still more time‐ and cost‐efficient in taxonomic groups for which just a few markers may be needed to infer well‐resolved phylogenies.

## APPENDIX 1 Collection locations, vouchers, and GenBank accessions for taxa included in this study.

**Table:**

Taxon^a^	Phylogenetic clade^b^	Collection locality	Voucher (Herbarium)^c^	GenBank accession no.		
				pgiC	IBR3	gapC
*Isoëtes butleri* Engelm.	Clade E	Texas, USA	*Schafran 47* (ODU)	KY243331	KY270816	KY270832
*I. echinospora* Durieu	Clade E	New York, USA	*Schafran NY‐4* (ODU)	KY243333	KY270818	KY270835
*I. engelmannii* A. Braun	Clade E	Tennessee, USA	*Schafran 46* (ODU)	KY243334	KY270819	—
*I. flaccida* Shuttlew. var. *chapmanii* Engelm.	Clade E( (= *I. flaccida*)	Florida, USA	*Bolin JB_FL_01* (ODU)	KY243332	KY270817	KY270833
*I. flaccida* var. *flaccida*	Clade E	Florida, USA	*Schafran FL‐01* (ODU)	KY243335	KY270820	KY270836
*I. histrix* Bory & Durieu	Clade E	Sicily, Italy	*A. Troia s.n*.^d^	KY243347	—	—
*I. lithophila* N. Pfeiff.	Clade E	Texas, USA	*Schafran 61* (ODU)	KY243336	KY270822	KY270838
*I. longissima* Bory	Clade B )(= *I. velata*)	Sicily, Italy	*A. Troia s.n*.^d^	KY243348	KY270823	KY270839
*I. melanopoda* J. Gay & Durieu subsp. *melanopoda*	Clade E	Mississippi, USA	*Taylor 6796* (US)	KY243338	KY270825	KY270841
*I. melanopoda* subsp. *silvatica* D. F. Brunt. & D. M. Britton	Clade E (= *I. melanopoda* s.l.)	North Carolina, USA	*Schafran NC‐05* (ODU)	KY243342	KY270828	KY270845
*I. melanospora* Engelm.	Clade E	Georgia, USA	*Schafran 12* (ODU)	KY243339	KY270826	KY270842
*I. nuttallii* A. Braun ex Engelm.	Clade B	California, USA	*Taylor 6734* (US)	KY243351	—	—
*I. piedmontana* (N. Pfeiff.) C. F. Reed	—	Georgia, USA	*Schafran 18* (ODU)	KY243341	KY270827	KY270844
*I. storkii* T. C. Palmer	Clade E	Costa Rica	*Taylor 6760* (US)	KY243352	KY270829	KY270846
*I. tegetiformans* Rury	—	Georgia, USA	*Schafran 19* (ODU)	KY243343	KY270830	KY270847
*I. valida* (Engelm.) Clute	Clade E	Pennsylvania, USA	*Schafran 37* (ODU)	KY243344	KY270831	—
*Lycopodium clavatum* L.	—	New York, USA	*Schafran s.n*.^e^	MG434746	—	—

Notes

^a^ One individual was sampled per taxon.

^b^ Per Larsén and Rydin (2016).

^c^ Herbaria are abbreviated according to Index Herbariorum (http://sweetgum.nybg.org/science/ih/).

^d^ Tissue samples provided by A. Troia (Università degli Studi di Palermo, Palermo, Italy); not deposited in a recognized herbarium.

^e^ Voucher deposited in P. Schafran's personal collection.

## APPENDIX 2 Amplification and sequence quality of markers across taxa.

**Table:**

Taxon	Amplification			Sequencing		
	pgiC	IBR3	gapC	pgiC	IBR3	gapC
*Isoëtes butleri*	+	+	+	+	+	+
*I. echinospora*	+	+	+	+	+	+
*I. engelmannii*	+	+	+	+	+	—
*I. flaccida* var. *chapmanii*	+	+	+	+	+	+
*I. flaccida* var. *flaccida*	+	+	+	+	+	+
*I. histrix*	+	—	—	+	NA	NA
*I. lithophila*	+	+	+	+	+	+
*I. longissima*	+	+	+	+	+	+
*I. melanopoda* subsp. *melanopoda*	+	+	+	+	+	+
*I. melanopoda* subsp. *silvatica*	+	+	+	+	+	+
*I. melanospora*	+	+	+	+	+	+
*I. nuttallii*	+	—	—	+	NA	NA
*I. piedmontana*	+	+	+	+	+	+
*I. storkii*	+	+	+	+	+	+
*I. tegetiformans*	+	+	+	+	+	+
*I. valida*	+	+	+	+	+	—
*Lycopodium clavatum*	+	—	+	+	NA	—

Note

+ = successful amplification or sequence quality >85%; — = no amplification or sequence quality <85%; NA = sequencing not attempted.

## APPENDIX 3 Pairwise number of nucleotide differences between pgiC/IBR3/gapC sequences.

**Table:**

	*I. butleri*	*I. echinospora*	*I. engelmannii*	*I. flaccida* var. *chapmanii*	*I. flaccida* var. *flaccida*	*I. histrix*	*I. lithophila*	*I. longissima*	*I. melanopoda* subsp*. melanopoda*	*I. melanopoda* subsp*. silvatica*	*I. melanospora*	*I. nuttallii*	*I. piedmontana*	*I. storkii*	*I. tegetiformans*	*I. valida*
*I. echinospora*	10/34/37															
*I. engelmannii*	4/30/—	8/26/—														
*I. flaccida* var*. chapmanii*	6/56/44	10/57/28	2/52/—													
*I. flaccida* var*. flaccida*	6/56/43	10/58/28	2/53/—	0/3/10												
*I. histrix*	73/—/—	78/—/—	72/—/—	70/—/—	70/—/—											
*I. lithophila*	4/41/51	6/36/40	2/39/—	4/68/45	4/69/44	79/—/—										
*I. longissima*	74/217/206	78/225/194	73/218/—	73/236/188	73/235/191	49/—/—	74/225/198									
*I. melanopoda* subsp*. melanopoda*	8/33/57	10/13/47	6/28/—	6/59/53	6/60/51	72/—/—	4/38/15	74/221/208								
*I. melanopoda* subsp*. silvatica*	8/35/35	11/36/26	4/28/—	6/61/31	6/62/29	74/—/—	6/40/37	74/225/198	10/38/44							
*I. melanospora*	9/26/47	12/23/32	5/16/—	7/47/37	7/48/36	75/—/—	7/34/30	75/218/201	11/25/36	1/27/37						
*I. nuttallii*	67/—/—	69/—/—	68/—/—	70/—/—	70/—/—	54/—/—	81/—/—	49/—/—	72/—/—	69/—/—	70/—/—					
*I. piedmontana*	8/26/40	11/23/14	4/16/—	6/47/30	6/48/27	74/—/—	6/34/42	74/218/191	10/25/44	0/27/25	1/2/36	69/—/—				
*I. storkii*	13/29/47	15/24/34	11/25/—	11/50/14	11/50/16	77/—/—	4/30/37	75/220/192	9/26/45	15/29/34	16/21/38	73/—/—	15/21/36			
*I. tegetiformans*	7/31/54	10/28/40	6/21/—	8/53/40	8/54/36	72/—/—	5/40/54	74/221/197	10/30/61	10/28/38	11/19/47	67/—/—	10/19/38	14/24/42		
*I. valida*	6/52/—	14/51/—	8/48/—	10/14/—	10/15/—	75/—/—	8/62/—	75/239/—	12/53/—	12/56/—	13/44/—	67/—/—	12/44/—	17/44/—	11/48/—	
*Lycopodium clavatum*	227/—/—	229/—/—	226/—/—	225/—/—	225/—/—	225/—/—	248/—/—	227/—/—	223/—/—	228/—/—	229/—/—	236/—/—	228/—/—	226/—/—	229/—/—	228/—/—
